# Restoration of Pulmonary Compliance after Laparoscopic Gynecologic Surgery Using a Recruitment Maneuver

**DOI:** 10.3390/jpm14050451

**Published:** 2024-04-25

**Authors:** Panagiota Griva, Christina Talliou, Loizos Rougeris, Dimitra Samara, Konstantina Panagouli, Giolanda Varvarousi, Maria Papa, Nikolaos Kathopoulis, Vasiliki Chantziara, Nikoletta Rovina

**Affiliations:** 1 Department of Anesthesiology, University General Hospital Attikon,12462 Athens, Greece; 2 Department of Anaesthesiology, Rea Maternity Hospital, 17564 Athens, Greece; 3 Department of Anaesthesiology, General Hospital of Athens Alexandra, 11528 Athens, Greece; 4 Department of Obstetrics and Gynaecology, General Hospital of Athens Alexandra, National and Kapodistrian University of Athens, 11528 Athens, Greece; 5 1st Department of Respiratory Medicine, Sotiria Thoracic Diseases Hospital of Athens, School of Medicine, National and Kapodistrian University of Athens, 11527 Athens, Greece

**Keywords:** pulmonary compliance, alveolar recruitment maneuver, laparoscopic procedures, gas exchange, pneumoperitoneum, Trendelenburg position

## Abstract

Background/Objectives: This study aimed to investigate the hypothesis that an alveolar recruitment maneuver can restore lung compliance to initial values after laparoscopic gynecological surgery. Methods: A total of 31 patients who underwent laparoscopic gynecological surgery were enrolled. Protective mechanical ventilation was applied, and the radial artery was catheterized in all patients. An alveolar recruitment maneuver (incremental and decremental positive end-expiratory pressure) was applied ten minutes after the release of pneumoperitoneum. The respiratory mechanics and blood gas results were recorded at eight different time points: after induction of anesthesia (T1), in the lithotomy position (T2), in the Trendelenburg position (T3), 10 and 90 min after insufflation of carbon dioxide (T4 and T5), in the supine position (T6), after desufflation (T7), and 10 min after an alveolar recruitment maneuver at the end of surgery (T8). Results: Pneumoperitoneum and the Trendelenburg position caused a decline of 15 units in compliance (T7 vs. T1; *p* < 0.05) compared to baseline. After the alveolar recruitment maneuver, compliance increased by 17.5% compared with the mean value of compliance at time T1 (T8 vs. T1; *p* < 0.05). The recruitment maneuver had favorable results in patients with low initial compliance (41.5 mL/cmH_2_O, IQR: 9.75 mL/cmH_2_O), high Body Mass Index 30.32 kg/m^2^ (IQR: 1.05 kg/m^2^), and high initial plateau airway pressure (16.5 cmH_2_O, IQR: 0.75 cmH_2_O). Conclusions: Lung compliance does not return to initial values after performing laparoscopic gynecological procedures. However, after the release of pneumoperitoneum, an alveolar recruitment maneuver is beneficial as it improves compliance and gas exchange.

## 1. Introduction

Minimally invasive surgery is considered the method of choice for surgical treatment of many gynecological diseases [1]. The first step in laparoscopic surgery is the induction of pneumoperitoneum. Gas insufflation is used to separate the abdominal wall from the intra-abdominal organs and achieve sufficient working space [2]. Different gases have been studied for the induction of pneumoperitoneum [3]. Carbon dioxide is the optimal choice as the risk of embolism is decreased [3]. The main advantages of laparoscopic surgery include a reduction in hospital stay, duration of operation, postoperative pain, and trauma due to the smaller surgical incision [4]. Some of the disadvantages are the higher cost, the technical difficulty in performing the operation, delay in bleeding control, tissue destruction, and nerve injury [5].

Laparoscopic procedures have a negative impact on the mechanical properties of the lung owing to pneumoperitoneum, changes in the patient’s position, and positive pressure mechanical ventilation [6,7]. Pneumoperitoneum and the Trendelenburg position affect respiratory system function [8]. Due to pneumoperitoneum, intra-abdominal pressure increases, resulting in the cranial displacement of the intra-abdominal organs. Elevation of the diaphragm leads to the collapse of the dependent lung areas [9], a decrease in respiratory system compliance, and an increase in peak inspiratory pressure and mean airway pressure [10]. Vital capacity and forced expiratory volume decreased in patients after laparoscopic surgery [11]. The Trendelenburg position during laparoscopic gynecological procedures enhances the effects of abdominal pressure through gravity and relaxation of the diaphragm. Thus, increased airway pressure and reduced functional residual capacity cause further changes in the respiratory system [12]. Intraoperative lung damage and the development of atelectasis affect postoperative lung function [13]. Failure to restore compliance predisposes patients to postoperative pulmonary complications [14]. The restoration of pulmonary compliance depends on several factors such as the duration of pneumoperitoneum and the position of the patient [14].

Concerning laparoscopic surgeries, lung ventilation can be a challenge for anesthesiologists. Carbon dioxide is reabsorbed from the abdominal cavity into circulation. The absorbed carbon dioxide is expelled from the lungs along with the carbon dioxide produced by cell metabolism. As the partial pressure of carbon dioxide is a determinant of acid–base balance, ventilation adjustment is necessary to avoid hypercapnia and respiratory acidosis. Minute ventilation must be increased by up to 15% to meet the respiratory demands [15].

Protective lung ventilation strategies, including low tidal volume (TV), positive end-expiratory pressure (PEEP), and a recruitment maneuver (RM), can reduce postoperative respiratory complications [16]. During an RM, airway pressure is increased for a short period of time in order to “open” collapsed alveoli and improve gas exchange and lung compliance [17]. An RM should last at least 7–8 s [18]. RM methods are divided into two categories. The first category includes an RM, which is performed by sustained inflation for 5–30s and defined peak inspiratory pressure (PIP). The second category is performed using a stepwise increase in the PEEP. Both methods involve the application of PEEP after RM to maintain the positive effects of recruitment [19]. An RM with a stepwise increase in PEEP and a constant driving pressure can recruit most of the atelectatic alveoli with minimal effects on the hemodynamic status and inflammatory response [20]. The most common complications include hypotension, desaturation, and bradycardia. These hemodynamic changes are transient and self-limiting. In a small percentage of patients (1%), an early cessation of RM is needed to achieve hemodynamic stability [21].

This study aimed to investigate the hypothesis that RMs restore lung compliance to baseline values after laparoscopic gynecological surgery. An RM was applied after pneumoperitoneum release and before extubation. After the RM, the atelectasis created intraoperatively might be reduced. As a result, postoperative respiratory complications are reduced. This is the first study that investigates the performance of a stepwise RM after desufflation during laparoscopic gynecologic procedures and the effect of the initial Pplat and DP on compliance intraoperatively.

## 2. Materials and Methods

The study protocol was approved by the Institutional Ethics Committee of General Hospital of Athens “Alexandra,” Athens, Greece, EU (No: 275/27-04-2023; date of approval: 25 May 2023; enrolment period: 7 June 2023 until 13 December 2023), registered at ClinicalTrials.gov registry (NCT: 06203665).

The study protocol was performed after obtaining written informed consent from each patient.

The inclusion criteria were patients older than 18 years, scheduled to undergo gynecological laparoscopic surgery, and with a minimum surgery duration of over 90 min. The exclusion criteria were patients under 18 years of age and Body Mass Index (BMI) over 32 kg/m^2^. Patients with chronic obstructive disease with FEV1 < 60% of the predicted volume, emphysema, right ventricular failure, and intraoperative hemodynamic instability (Mean Arterial Pressure < 65 mm Hg) were excluded.

### 2.1. Anesthetic Management

Following fasting guidelines, patients did not consume solid foods or clear liquids after six and two hours, respectively. In the operating room, an 18G or 20G venous catheter was inserted and patients were monitored with electrocardiography (ECG), pulse oximetry, and noninvasive blood pressure measurement. Ideal body weight was calculated according to the formula x + 2.3 kg for every 2.54 cm above 152.4 cm height; x = 45.5 for women [14]. The pharmacological doses and ventilator settings were adjusted based on this calculation.

For induction to anesthesia, propofol 1.5–2.5 mg/kg, a neuromuscular blocker (rocuronium 0.6 mg/kg), and opioids (fentanyl) were administered. A Carestation 650 ventilator (GE Healthcare) was used for the mechanical support of breathing. After endotracheal intubation, protective mechanical ventilation was applied: tidal volume (Vt) of 6–8 mL/kg of ideal weight, PEEP = 5 cm H_2_O, respiratory rate, and I:E ratio were adjusted accordingly to maintain end-tidal carbon dioxide (EtCO_2_) levels between 35 and 45 mmHg. Anesthesia was maintained by administration of the volatile anesthetic desflurane (6–6.5 vol%) to achieve a minimal alveolar concentration (MAC) of 1. After performing the Allen test, an arterial line catheter was placed in the radial artery. Arterial gas analysis was performed using a GEM Premier 3500 system with an Intelligent Quality Management (Iqm) analyzer. Mean insufflation pressure 10 and 90 min after pneumoperitoneum induction was 13.3 ± 2.9 mmHg and 12.5 ± 2.1 mmHg, respectively. The inclination in the Trendelenburg position was 15–20°. Sugammandex (2 mg/kg) was administered to all patients. In the post-anesthesia care unit (PACU), oxygen was administered to all patients using a nasal cannula at a flow rate of 2 L/min.

### 2.2. Study Protocol

Mechanical properties of the lung (Compliance), peak inspiratory pressure (PIP), end-inspiratory pressure (plateau airway pressure (Pplat)), and resistance [airway resistance (Raw)] were recorded during the operation. Mechanical properties of the lung along with arterial blood gas results were recorded at the following time points: after induction of anesthesia (T1), lithotomy position (T2), Trendelenburg position (T3), 10 and 90 min after pneumoperitoneum induction (T4 and T5), supine position (T6), after desufflation (T6 and T7), and 10 min after RM (T8). Incremental and decremental positive end-expiratory pressure RMs were performed five minutes after releasing pneumoperitoneum, as long as hemodynamic stability was ensured (Mean Arterial Pressure > 65 mmHg, Heart Rate > 60 bpm). An RM was performed in a pressure-controlled ventilation model with an inspiratory pressure of 15 cmH_2_O, PEEP of 5 cmH_2_O, FiO_2_ of 1.0, number of breaths of 10 breaths/min, and I/E ratio of 1:1. PEEP was increased by 5 cmH_2_O every five breaths until the peak airway pressure was 40 cmH_2_O. When the maximum airway pressure was 40 cmH_2_O, ventilation was maintained for two minutes and then PEEP was decreased by 2 cmH_2_O every three breaths. Respiratory rate and FiO_2_ were adjusted to achieve adequate ventilation.

### 2.3. Outcome

#### 2.3.1. Primary Outcome

To evaluate the primary outcome, the compliance difference between T1 and T7 [Delta (Δ) Compliance (T1–T7)] was calculated. The compliance difference between T1 and T8 [Delta (Δ) Compliance (T1–T8)] was also calculated, and the difference in compliance between T7 and T8 [Delta (Δ) Compliance (T7–T8)]. 

#### 2.3.2. Secondary Outcomes

Dynamic compliance (Cdyn, mL/cmH_2_O), end-inspiratory airway pressure (Pplat, cmH_2_O), and driving pressure (DP, cmH_2_O) were recorded at the aforementioned time points (T1–T8), and arterial blood gases were also measured. The parameters recorded were pH, PaO_2_ (mmHg), PCO_2_ (mmHg), and HCO_3_ (mEq/L).

To assess hemodynamic variables, Mean Arterial Pressure (MAP) and Heart Rate (HR) were monitored every three minutes.

Other recorded data included surgery duration, duration of anesthesia, intraoperative fluids, blood loss, and intraoperative diuresis. Postoperative complications (pulmonary, cardiac, renal) were recorded.

### 2.4. Sample Size Calculation

The null hypothesis was that RMs could restore pulmonary compliance to the initial values. Using GPower 3.1.2 for Windows (Duesseldorf, Germany), the power of the study was set to 80%, the type I error probability (α) was 0.05, and a minimal sample size of 28 patients was required to obtain statistically significant results. A total of 31 patients were enrolled in this study, considering possible losses.

### 2.5. Statistical Analysis

Statistical analysis was performed using the programming language Python. Mann–Whitney U test was applied to explore potential statistically significant variations in the sample. The Mann–Whitney U test is appropriate for statistical data that do not follow a normal distribution, which is the case for the dataset of this study. The Kolmogorov–Smirnov test was utilized to evaluate the dataset’s distributional properties. As depicted in Table S5 (Supplementary Material), there was no evidence of statistical significance in the variability of the data among the 31 patients (*p* > 0.05, where 0.05 represents the chosen alpha value for each of the investigated parameters). The absence of statistical significance emphasized the homogeneity observed across the patient dataset. These results provide valuable insights into the uniformity of the conditions within the investigated protocol. Furthermore, the absence of statistical significance serves as a robust foundation for the stratification analysis conducted in the next step of this work. The primary objective of this protocol was to derive valuable insights from assessing the effectiveness of recruitment maneuvers using various patient parameters and other specific features. Consequently, stratification analysis was deemed the most effective approach for attaining these goals. Specifically, the analysis focused on the following parameters: compliance at T1 (Cdyn_T1), Plateau Pressure at T1 (Pplat_T1), and Body Mass Index (BMI). For each of these parameters, the dataset of 31 patients was stratified into two groups: one comprising 10 patients with the highest values of the examined parameter and the other with 10 patients exhibiting the lowest respective values. Subsequently, the statistical significance of all parameters was assessed using the Mann–Whitney U test to determine whether any observed differences could be attributed to variations in the examined parameters.

## 3. Results

The demographic characteristics of the patients are presented in Table 1. Characteristics of the procedures are depicted in Table 2.

**Figure 1:**
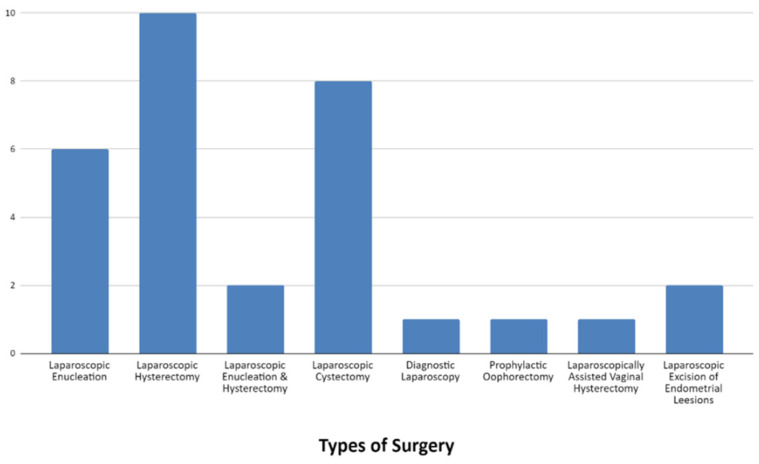
Types of surgery.

**Figure 2:**
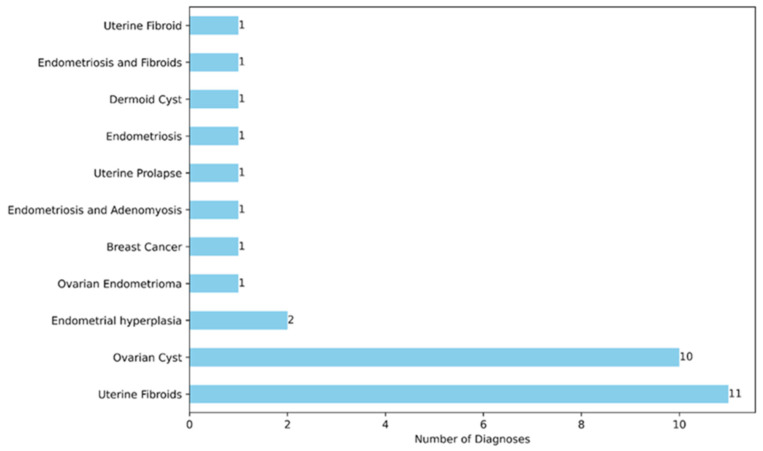
Distribution of diagnoses.

The study protocol was successfully applied in each patient without complications. Data are presented as the median values. The different types of surgery and diagnoses are shown in Figure 1 and Figure 2, respectively.

**Figure 3:**
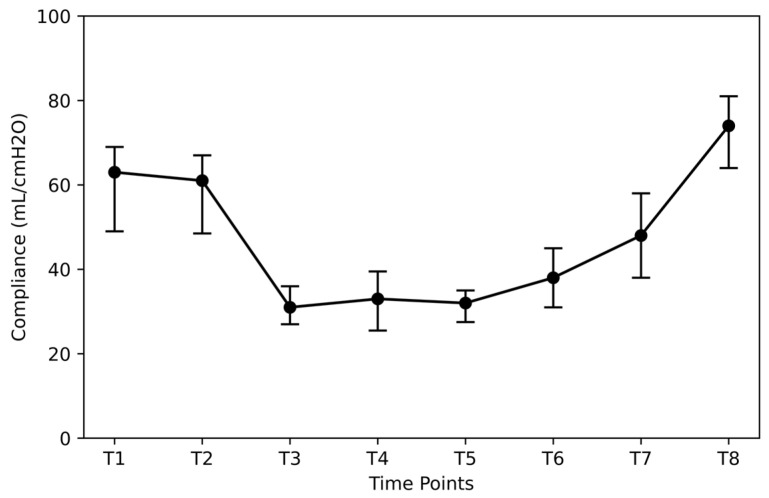
Compliance at different time points. Time points: after induction of anesthesia (T1), in the lithotomy position (T2), in the Trendelenburg position (T3), 10 and 90 min after insufflation of carbon dioxide (T4 and T5), in the supine position (T7), after desufflation (T7), and 10 min after an alveolar recruitment maneuver at the end of surgery (T8).

After the induction of anesthesia (T1), the median value of lung compliance was 63.0 mL/cmH_2_O (IQR = 20.0 mL/cmH_2_O) (Figure 3). When patients were placed in the lithotomy position (T2), the median compliance value showed a slight decline of 3.2% (T2 vs. T1; *p* < 0.05). When placed in the Trendelenburg position (T3), the median compliance decreased by 50.8% compared to the baseline (T3 vs. T1; *p* < 0.05). A further reduction of 47.6% (T4 vs. T1; *p* < 0.05) occurred after insufflation (T4). The lowest observed median compliance value was 31.0 mL/cmH_2_O (IQR = 7.5 mL/cmH_2_O) and was recorded 90 min after insufflation (T5). After placing the patient in the neutral position (T6), the median compliance was increased by 22.6% from the corresponding value at the time point T5 (T6 vs. T5; *p* < 0.05). However, it remained 39.7% lower than that at baseline at T1 (T6 vs. T1; *p* < 0.05). After desufflation (T7), compliance elevated to 48.0 mL/cmH_2_O (IQR = 20.0 mL/cmH_2_O) but remained lower than T1 by 15 units (T7 vs. T1; *p* < 0.05). After RM, the median value of compliance was 74.0 mL/cmH_2_O (IQR = 17.0 mL/cmH_2_O). The increase corresponds to a percentage of 18% compared to the median value of compliance at the time point T1 (T8 vs. T1; *p* < 0.05).

**Figure 4:**
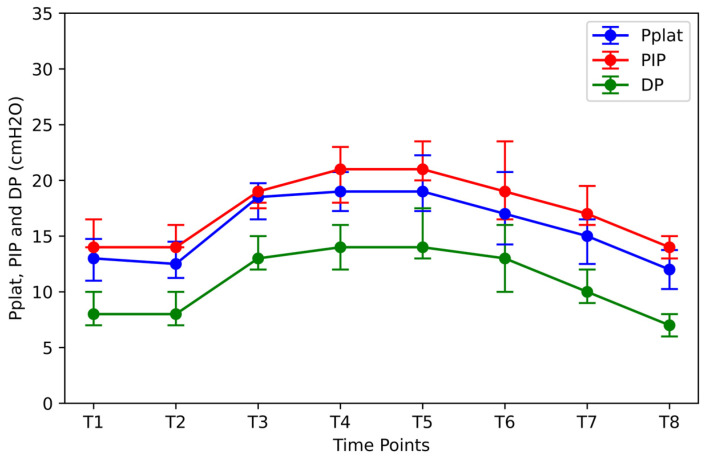
Pplat, PIP, and DP at different time points. Time points: after induction of anesthesia (T1), in the lithotomy position (T2), in the Trendelenburg position (T3), 10 and 90 min after insufflation of carbon dioxide (T4 and T5), in the supine position (T7), after desufflation (T7) and 10 min after an alveolar recruitment maneuver at the end of surgery (T8). Pplat = end-inspiratory pressure; PIP = peak inspiratory pressure; DP = driving pressure.

Pplat reached its maximum value at T5 (Figure 4). This increase was 46.1% (T5 vs. T1; *p* < 0.05). When the patients were placed in the supine position (T6) and after desufflation (T7), the median Pplat value gradually decreased but remained elevated compared to the initial value by 30.8% (T6 vs. T1; *p* < 0.05) and 15.4% (T7 vs. T1; *p* < 0.05), respectively. After RM (T8), Pplat was further reduced by 7.7% compared to the initial value at T1 (T8 vs. T1; *p* < 0.05).

Driving pressure (DP) and peak inspiratory pressure (PIP) showed similar elevation tendencies (Figure 4). At time point T5, they showed maximum increases of 75.0% and 50.0%, respectively (T5 vs. T1; *p* < 0, 05). By returning to the supine position and removing pneumoperitoneum, a decrease in the average value was observed. However, both the median DP and median PIP remained elevated relative to their initial values at T1 by 25.0% and 21.4%, respectively (T7 vs. T1; *p* < 0.05). After RM (T8), the median DP showed a decline of 12.5% compared to its median value at T1 (T8 vs. T1; *p* < 0.05). Median PIP returned to the baseline values.

**Figure 5:**
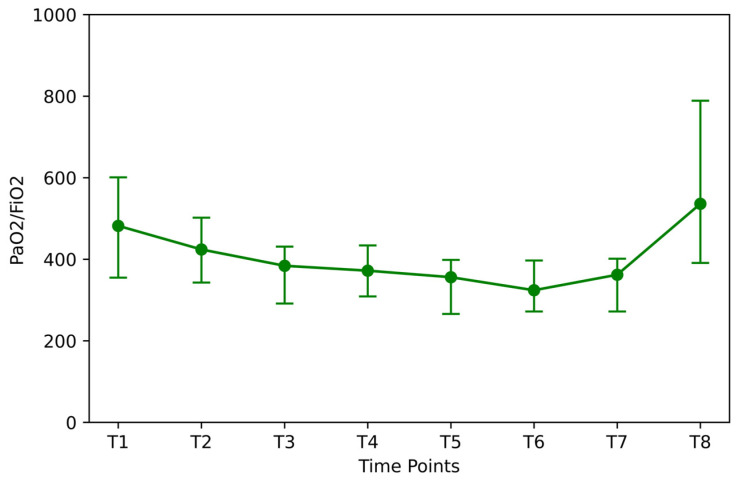
PaO_2_/FiO_2_ ratio at different time points. Time points: after induction of anesthesia (T1), in the lithotomy position (T2), in the Trendelenburg position (T3), 10 and 90 min after insufflation of carbon dioxide (T4 and T5), in the supine position (T7), after desufflation (T7), and 10 min after an alveolar recruitment maneuver at the end of surgery (T8). PaO_2_ = partial pressure of oxygen; FiO_2_ = Inspiratory Oxygen.

After the induction of anesthesia, the initial PaO_2_/FiO_2_ ratio was 482.0 ± 246.0 (Figure 5), and it reduced by 12.0% in the lithotomy position (T2 vs. T1; *p* < 0.05). After the Trendelenburg position and insufflation, an additional 22.9% drop was recorded (T4 vs. T1; *p* < 0.05). Ninety minutes after insufflation (T5), the ratio showed a 27% percentage decrease compared with the initial value (T5 vs. T1; *p* < 0.05). After desufflation, the ratio increased by 38 units (T6 vs. T7; *p* < 0.05) but remained 24.9% lower than the baseline (T7 vs. T1; *p* < 0.05). After RM, the PO_2_/FiO_2_ ratio returned to the baseline values (T7 vs. T1; *p* < 0.05).

**Figure 6:**
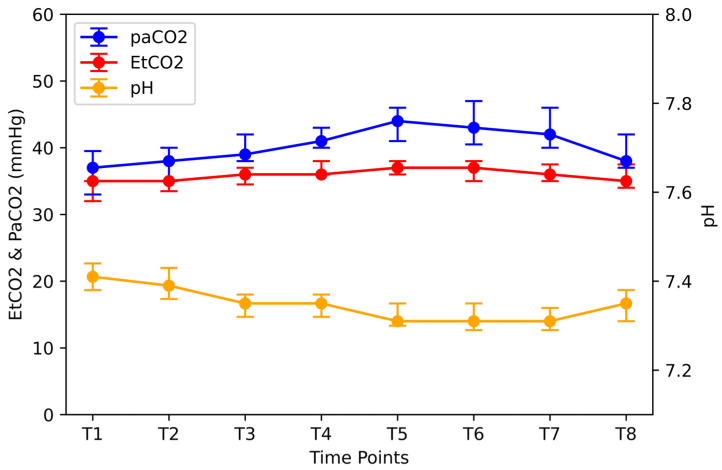
PaCO_2_, EtCO_2_, and pH at different time points. Time points: after induction of anesthesia (T1), lithotomy position (T2), Trendelenburg position (T3), 10 and 90 min after CO_2_ insufflation (T4 and T5), immediately after lithotomy removal and after pneumoperitoneum removal (T6 and T7), and 10 min after an alveolar recruitment manipulation at the end of surgery (T8). PaCO_2_ = partial pressure of carbon dioxide; EtCO_2_ = end-expiratory pressure of carbon dioxide.

pH declined significantly during surgery compared to baseline (T7 vs. T1; *p* < 0.05) (Figure 6) but returned to initial values after RM (T8 vs. T1; *p* < 0.05). Acidosis was caused by an increase in PaCO_2_ (Figure 6). Specifically, the percentage increase during surgery was 19% (T5 versus T1; *p* <0.05). After the release of pneumoperitoneum (T7) and RM (T8), the mean PaCO_2_ decreased by 2.3% (T7 vs. T8; *p* < 0.05) and remained elevated by 2.7% compared with the baseline value (T8 vs. T1; *p* < 0.05).

**Figure 7:**
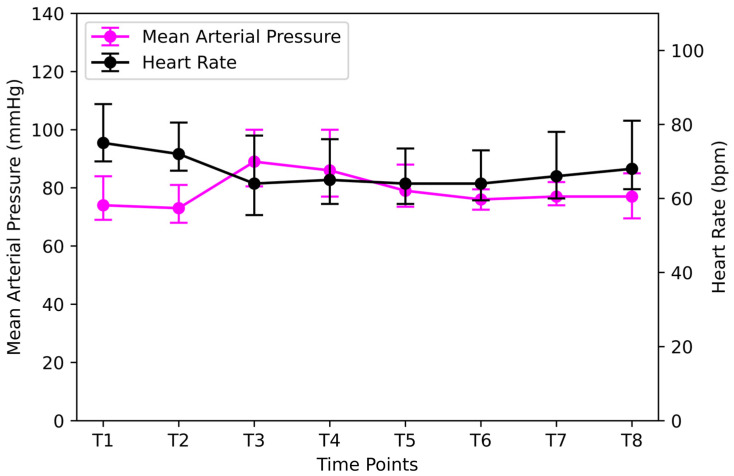
MAP and HR at different time points. Time points: after induction of anesthesia (T1), lithotomy position (T2), Trendelenburg position (T3), 10 and 90 min after CO_2_ insufflation (T4 and T5), immediately after lithotomy removal and after pneumoperitoneum removal (T6 and T7), and 10 min after an alveolar recruitment manipulation at the end of surgery (T8). MAP = Mean Arterial Pressure; HR = Heart Rate.

MAP and HR remained relatively stable throughout the protocol (Figure 7). Ephedrine was the vasoconstrictor of choice because of its positive chronotropic and inotropic effects. To maintain the MAP above 65 mmHg during RM, an average of 12.1 mg of ephedrine was administered. Notably, the maximum dose of ephedrine administered was 50 mg. The patient developed severe bradycardia (Heart Rate 36 beats/min) during the RM.

**Figure 8:**
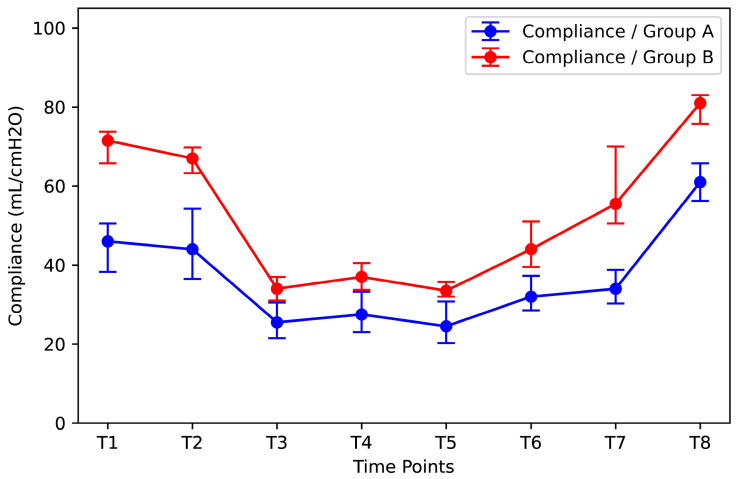
Effect of the initial Pplat on compliance at different time points. Group A: the group of patients with the highest initial Pplat values (median: 16.5 cmH_2_O, IQR: 0.75 cmH_2_O); Group B: the group of patients with the lowest initial Pplat values (median: 11.0 cmH_2_O, IQR: 4.5cmH_2_O). Pplat = end-inspiratory pressure.

Patients were divided into two groups (Group A and Group B) according to Pplat at time T1. Group A included the patients with the highest initial Pplat values (median: 16.5 cmH_2_O, IQR: 0.75 cmH_2_O). Group B included patients with the lowest Pplat values (median: 11.0 cmH_2_O, IQR: 4.5cmH_2_O). The compliance of Group B was significantly higher than that of Group A at all time points (Figure 8). Although RM significantly improved compliance in Group A [increase from 34 mL/cmH_2_O (IQR = 8.5 mL/cmH_2_O) at time T7 to 61.00 mL/cmH_2_O (IQR = 9.50 mL/cmH_2_O) at time T8], it remained statistically significantly lower than the corresponding value of Group B [increase from 55.5 mL/cmH_2_O (IQR = 19.5 mL/cmH_2_O) at time T7 to 81.0 mL/cmH_2_O (IQR = 7.25 mL/cmH_2_O) at time T8].

**Figure 9:**
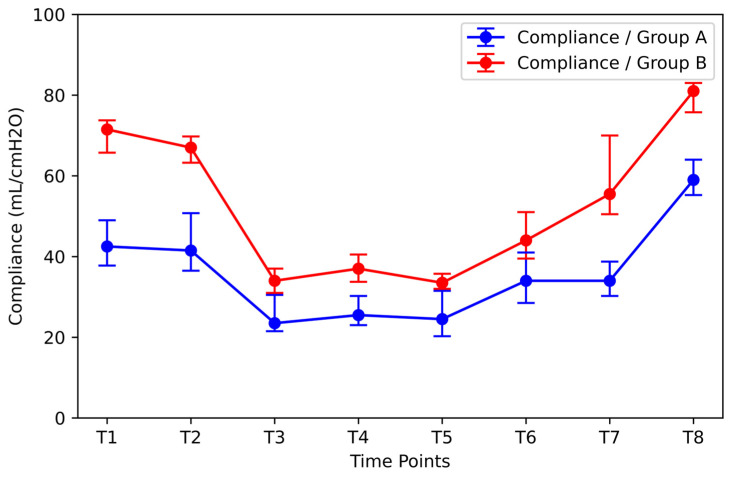
Effect of initial DP on compliance. Group A: the group of patients with the highest initial DP values (median: 11.5 cmH_2_O, IQR: 4.5 cmH_2_O); Group B: the group of patients with the lowest initial DP values (median: 6.0 cmH_2_O, IQR: 0.75 cmH_2_O). DP = driving pressure.

The same methodology was used to investigate the effect of the initial DP on compliance (Figure 9). Group A included patients with the highest initial DP values (median: 11.5 cmH_2_O, IQR: 4.5 cmH_2_O). Group B included patients with the lowest DP values (median: 6.0 cmH_2_O, IQR: 0.75 cmH_2_O). Compliance was significantly higher than in Group A time points (T1–T8). After RM, the compliance of Group A patients increased significantly [increase from 13 cmH_2_O (IQR = 4.25 cmH_2_O) at time T7 to 13 cmH_2_O (IQR = 4.25 cmH_2_O) at time T8] but remained statistically significantly lower than the corresponding value of Group B [increase from 8.5 cmH_2_O (IQR = 2.75 cmH_2_O) at time T7 to 6 cmH_2_O (IQR = 1.75 cmH_2_O) at time T8].

**Figure 10:**
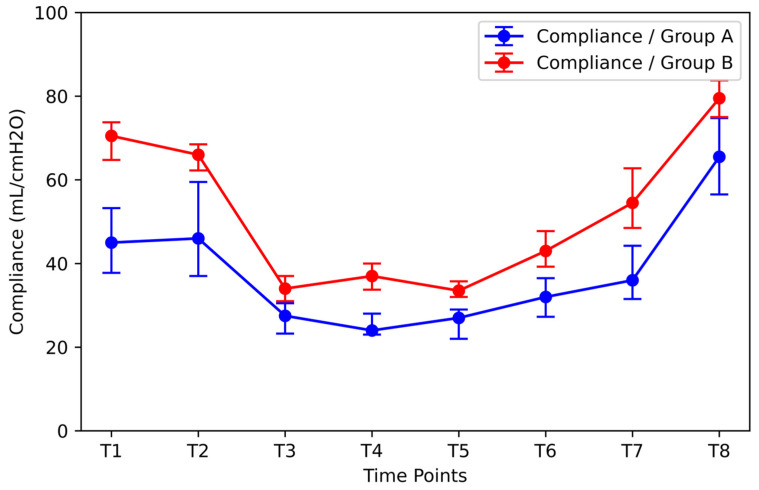
Effect of initial BMI on compliance. Group A: the group of patients with the highest BMI values (median: 30.32 kg/m^2^, IQR: 1.05 kg/m^2^); Group B: the group of patients with the lowest BMI values (median: 21.28 kg/m^2^, IQR: 0.39 kg/m^2^). BMI = Body Mass Index.

The results of the effect of BMI on compliance are shown in Figure 10. The median BMI value for Group A patients was 30.32 kg/m^2^ (IQR: 1.05 kg/m^2^), while for those in Group B, it was 21.28 kg/m^2^ (IQR: 0.39 kg/m^2^). Patients with the lowest median BMI showed significantly higher compliance values compared to those in Group A at time points T1 to T7. This difference was not statistically significant at T8. Patients with a higher BMI showed a 79.4% increase between T7 and T8, which was significantly higher than that in Group B (46%).

**Figure 11:**
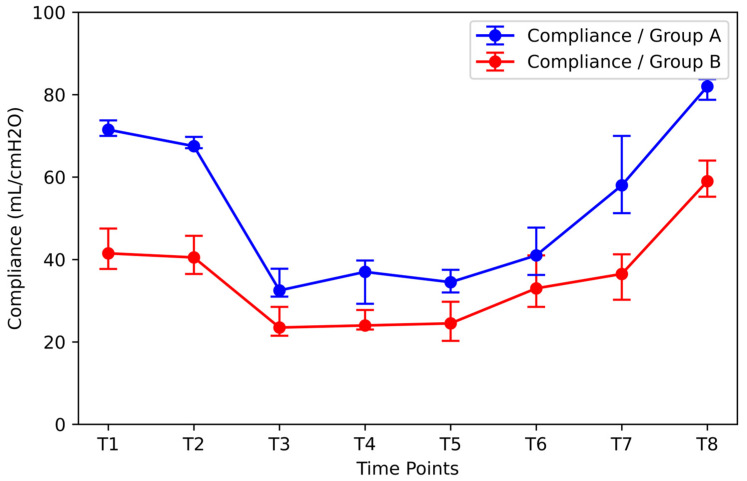
Effect of compliance. Group A: the group of patients with the highest initial compliance values (median: 71.5 mL/cmH_2_O; IQR: 3.75 mL/cmH_2_O); Group B: the group of patients with the lowest initial compliance values (median: 41.5 mL/cmH_2_O; IQR: 9.75 mL/cmH_2_O).

Initial compliance was evaluated and its effect on compliance variation during surgery (Figure 11). Group A included patients with the highest initial compliance values (median: 71.5 mL/cmH_2_O; IQR: 3.75 mL/cmH_2_O). Group B included patients with the lowest compliance values (41.5 mL/cmH_2_O; IQR: 9.75 mL/cmH_2_O). The difference in BMI between the two groups of patients was statistically significant [median: 21.39 kg/m^2^, IQR: 0.7 kg/m^2^ for Group A vs. 28.62 kg/m^2^, IQR: 3.18 kg/m^2^ for Group B). The compliance values of Group A were significantly higher than those of Group B at all time points except T6. After RM, the compliance of Group B patients increased significantly (increase from 36.5 mL/cmH_2_O, IQR: 11 mL/cmH_2_O at time T7 to 59 mL/cmH_2_O, IQR: 8.75 mL/cmH_2_O at time T8), remaining statistically significantly lower than the corresponding value of Group A (increase from 58 mL/cmH_2_O, IQR: 18.75 mL/cmH_2_O at time T7 to 82 mL/cmH_2_O, IQR: 5.0 mL/cmH_2_O at time T8).

**Figure 12:**
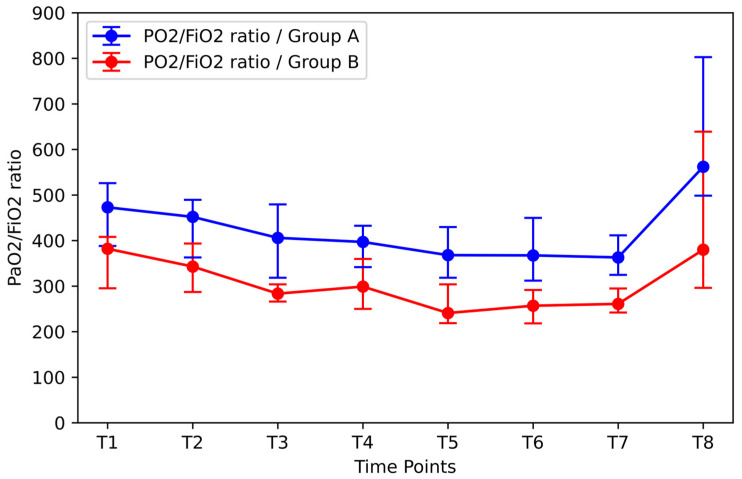
Effect of initial compliance on PaO_2_/FiO_2_ ratio. Group A: The group of patients with the highest initial compliance values (median: 71.5 mL/cmH_2_O; IQR: 3.75 mL/cmH_2_O) Group B: The group of patients with the lowest initial compliance values (median: 41.5 mL/cmH_2_O; IQR: 9.75 mL/cmH_2_O).

Initial compliance values significantly affected changes in the PaO_2_/FiO_2_ ratio (Figure 12). Patients with higher initial compliance showed significantly higher PaO_2_/FiO_2_ values for T2 to T7 compared to the group of patients with the lowest mean initial compliance. The PaO_2_/FiO_2_ ratio values showed a decrease of 23.3% in Group A and 31.7% in Group B between T1 and T7. The PaO_2_/FiO_2_ ratio remained below the initial value after desufflation. Subsequently, after the end of the RM, the PaO_2_/FiO_2_ values increased in both groups. For patients with a comparatively lower initial compliance, the percentage increase in the ratio between T7 and T8 was 45.6%, while the corresponding percentage increase for patients with a higher initial compliance value was 54.9%.

Lung compliance is significantly reduced during laparoscopic gynecological procedures due to pneumoperitoneum, as well as the Trendelenburg position. As shown in this study, despite the removal of these factors, the compliance remained 23.8% lower than the initial value at the beginning of the operation. With the RM, compliance returned to the initial values. Specifically, it increased by 17.46% compared to the compliance during induction of anesthesia. Moreover, patients with an initial median Pplat of 16.5 cmH_2_O (IQR: 0.75 cmH_2_O) benefit from RMs in comparison with the patients with an initial median Pplat of 11.0 cmH_2_O (IQR: 4.5cmH_2_O). Correspondingly, in patients with an initial median DP of 11.5 cmH_2_O (IQR: 4.5 cmH_2_O), an RM is more effective. Concerning the group of patients with a median BMI of 30.32 kg/m^2^ (IQR: 1.05 kg/m^2^), an RM was more beneficial compared with the group of patients with a median BMI of 21.28 kg/m^2^ (IQR: 0.39 kg/m^2^).

Patients with a lower MAP intraoperatively required ephedrine administration during the RM in order to maintain MAP above 65 mmHg. The median dose of ephedrine that was administrated was 10 mg vbg. The duration of surgery had no statistically significant effect on compliance and Pplat. Additionally, the average fluid administration rate of 10.6 mL/kg/h did not statistically significantly affect compliance.

## 4. Discussion

This study demonstrated that compliance does not return to initial values after desufflation, but it can be restored by applying an alveolar recruitment maneuver at the end of gynecological procedures. Specifically, after desufflation, median compliance remained 15 mL/cmH_2_O units lower than the baseline median value. After an RM, compliance increased by 17.5% compared with the initial average value.

During laparoscopic surgery, the increase in intra-abdominal pressure distends the abdominal wall by expanding its elasticity and causes cephalic displacement of the diaphragm [22]. Therefore, the pressure applied to the dependent areas of the lung increases, resulting in deterioration of the mechanics of the respiratory system. In this study, compliance decreased by 46.4% after pneumoperitoneum insufflation. During an RM, the airway pressure rises temporarily to recruit the alveoli that collapse during surgery. Consequently, the “open” alveolar unit participates in ventilation and gas exchange, resulting in improved oxygenation. The RM was beneficial because compliance improved by 54.2% (T7 vs. T8; *p* < 0.05) and the PaO_2_/FiO_2_ ratio increased by 46.4% (T7 vs. T8; *p* < 0.05).

The restoration of compliance after RM that was shown in this study agrees with the already existing research on this subject. Pei et al. [23] conducted a meta-analysis encompassing seven studies involving 628 patients, where static compliance measurements were documented. This analysis revealed the advantageous role of RMs in lung compliance. In a previous study by Cakmakkaya et al. [24], compliance remained at 10 cmH_2_O less than the baseline values after desufflation during laparoscopic nephrectomy. After the RM, it returned to the initial values. In the study by Sümer et al. [25], patients who underwent laparoscopic sleeve gastrectomy were divided into two groups. In one group, an RM was not performed (non-RM group), whereas in the other group, it was conducted five minutes after desufflation (RM group). It was reported that lung compliance was 18% higher in the RM group than in the non-RM group. The results of the study by Cinella et al. [8] showed that the elasticity of the chest wall exhibited a 30% increase, while the elasticity of the lung showed a 20% increase after RM. Based on statistical stratification analysis on the effect of initial Pplat (T1) on the patient’s compliance during the surgery (T1–T8), the group of patients with high initial Pplat (median: 16.5 cmH_2_O, IQR: 0.75 cmH_2_O) showed a median compliance of 46.0 mL/cmH_2_O, IQR: 12.25 mL/cmH_2_O after the induction of anesthesia. Compliance was 33.7% lower than that in the group of patients with low initial Pplat (median: 11.0 cmH_2_O, IQR: 4.5cmH_2_O). Between the two groups, the RM was more effective in patients with a higher baseline Pplat. It is worth noting that the median compliance value of the patients with a high initial Pplat at time T8 was 61.0 mL/cmH_2_O (IQR: 9.5 mL/cmH_2_O), approximately equal to the compliance of the patients with a lower initial Pplat at time T7 (55.5 mL/cmH_2_O; IQR: 19.5 mL/cmH_2_O). This justifies that RM was required for high-baseline-Pplat patients to reach the same level of compliance as lower-baseline-Pplat patients.

The type of RM that is used more often in the existing literature is sustained lung inflation, whereas, in this study, the RM that was performed was a stepwise RM. To perform an RM, Cakmakkaya et al. [24] used sustained lung inflation to 40 cmH_2_O for 10 s. After the RM, they observed a significant increase in mean oxygen pressure (PaO2). Accordingly, in the study by Sümer et al. [25], increasing the airway pressure to 40 cmH_2_O for 40 s resulted in a significant increase in PaO2. Sustained inspiratory pressure at 35 cmH_2_O for 20 s was the RM used by Oh et al. [26] in laparoscopic prostatectomies. The PaO_2_/FiO_2_ ratio improved by 39.3% after the RM in the lateral position. A stepwise RM was applied in the study by Cinnella et al. [8]. In the present study, incremental and decremental positive end-expiratory pressures were applied, as described above. This stepwise elevation of PEEP and constant guide pressure recruit more of the collapsed lung, minimizing hemodynamic effects and inflammation [27]. In addition, this stepwise RM without a prolonged increase in airway pressure is associated with fewer biological effects on the lungs [28]. Indeed, considerable augmentation of the PaO_2_/FiO_2_ ratio (46.4%, T7 vs. T8; *p* < 0.05) was observed after the RM.

Because an RM is a form of mechanical ventilation in which a high volume and high pressure are applied, significant hemodynamic instability may occur. During an RM, high airway pressure is transmitted through the lung parenchyma to the pleural space. As a result, the venous return and cardiac output are reduced. High alveolar pressure causes a concomitant increase in pulmonary vascular resistance, thereby increasing right ventricular afterload. Because of this increase in afterload, the ventricular septum shifts to the left, increasing the left ventricular end-diastolic filling pressure and decreasing left ventricular compliance, resulting in a further reduction in cardiac output. Cardiac compliance is reduced owing to the direct pressure applied to the cardiac cavity because of the large inspiratory volumes [29]. The stretch of the lung tissue caused by hyperdistension can activate the vagus reflex, leading to bradycardia. In a study by Cinella et al. [8], during an RM, the cardiac index (CI) decreased by 20%. In the present study, an average dose of 10.0 mg ephedrine was administered with the aim of maintaining MAP above 65 mmHg.

General anesthesia can lead to the collapse of 10–15% of lung tissue [30]. Τhe notable difference between this study and the one by Cinella et al. [8] is the time point of the surgery where the RM was performed. The study by Cinnella et al. [8] included 29 women who underwent laparoscopic surgery. An RM was performed 15 min after insufflation as long as the patients remained hemodynamically stable (MAP > 80 mmHg, HR > 60 bpm). As a result, chest wall elastance and oxygenation improved. They reported that the PaO_2_/FiO_2_ ratio increased by 5.8% before and after the RM. In this study, an RM was performed 5 min after desufflation. The PaO_2_/FiO_2_ ratio demonstrated an increase of 63.8% before and after the RM. Patel et al. [30] reported that even in short-term pneumoperitoneum (15–80 min), atelectases were found in the dependent part of the lung, as depicted on lung ultrasound intraoperatively. In a study by Monastesse et al. [31], prolonged pneumoperitoneum was associated with an increased loss of aerated alveoli. In accordance with the literature [30,31], the RM was conducted post-desufflation to avoid the development of a new atelectasis in case it was performed at an earlier time point.

The results of this study concerning overweight patients are in accordance with recent studies on obese patients and the alternations in their respiratory mechanics. Lung compliance and chest wall compliance are reduced in obese patients, mainly owing to the deposition of fat tissue in the mediastinum and abdominal cavity [32]. Morbidly obese patients have a reduced residual vital capacity in the supine position, which is further reduced by pneumoperitoneum and the Trendelenburg position. Insufflation of carbon dioxide represents an increased burden on the respiratory system owing to an increase in intra-abdominal pressure [33]. Obese patients are prone to developing intraoperative atelectasis. Improvement of atelectasis is delayed in obese patients compared with non-obese patients [34]. The results of relevant literature investigations [16,25] have shown that RMs and PEEP in morbidly obese patients undergoing laparoscopic operations are effective measures to improve lung mechanical properties and oxygenation. In the present study, patients with a median BMI of 30.32 kg/m^2^ (IQR: 1.05 kg/m^2^) showed a median compliance of 45.0 mL/cmH_2_O (IQR: 15.5 mL/cmH_2_O) during T1, while the corresponding median value in patients with a median BMI of 21.28 kg/m^2^ (IQR: 0.39 kg/m^2^) was 70.5 mL/cmH_2_O (IQR: 9.0 mL/cmH_2_O), i.e., 36.2% higher. However, after RM (T8), the percentage difference in compliance between the two groups of patients decreased to 17.6%. This is due to the fact that between T7 and T8, the increase in compliance in patients with a BMI of 30.32 kg/m^2^ (IQR: 1.05 kg/m^2^) was 81.9%, compared to a 45.9% augmentation in patients with a BMI of 21.28 kg/m^2^ (IQR: 0.39 kg/m^2^). Therefore, the RM was more efficient in patients with a higher BMI. Patients with a lower BMI exhibited significantly lower median Pplat at T1 (12.0 cmH_2_O, IQR: 2 cmH_2_O) compared to those with a higher initial BMI, who demonstrated a higher corresponding median value (15.0 cmH_2_O, IQR: 6.5 cmH_2_O). Additionally, a 34.2% reduction in Pplat between T7 and T8 was noted for patients with a BMI of 30.32 kg/m^2^ (IQR: 1.05 kg/m^2^), while a 17.9% decrease in patients with a BMI of 21.28 kg/m^2^ (IQR: 0.39 kg/m^2^) was observed. This also demonstrates the effectiveness of RM in patients with higher BMI.

The correlation between lung compliance and fluid administration has not been investigated. Therefore, no relevant references are available. Fluid overload has a negative impact on respiratory function owing to the accumulation of fluid in the interstitial space. The presence of excess fluid in the lungs affects gas exchange, reduces pulmonary compliance, and increases the work of breathing [35]. Regarding laparoscopic procedures, recent meta-analyses evaluating goal-directed and non-directed fluid administration do not provide recommendations for fluid administration in such types of surgeries [36]. In a study by Cinnella et al. [8], normal saline was administered at a dose of 8 mL/kg before the induction of anesthesia and was infused intraoperatively at a flow rate of 5 mL/kg/h. Continuous infusion of lactated Ringer’s solution at a flow rate of 5–10 mL/kg/h was administered intraoperatively during a study by Cakmakkaya et al. [24]. Sümer et al. [25] administered targeted fluids to avoid hypovolemia. In the present study, the patients received an average of 10.6 mL/kg/h. No statistically significant association was observed between lung compliance and fluid administration.

This study had several limitations. Healthy women (ASA class I or II) who underwent elective surgery were enrolled in this study. Therefore, the impact of the RM on the mechanical properties of the lungs in patients with pre-existing cardiopulmonary disease should be investigated in future studies. Second, patients with a BMI greater than 32 kg/m^2^ were excluded from this study. Therefore, further studies should investigate whether an RM is beneficial in such cases. Third, this study involved only women. It would be of scientific interest if future studies include men and other types of surgeries. Fourth, the sample size was from a single center. Multicenter studies should be performed to confirm these results. Finally, in the present study, the RM was performed in a stepwise manner (incremental and decremental positive end-expiratory pressures). It is suggested to investigate different types of RMs in order to identify any differences in their effectiveness in opening collapsed alveoli.

## 5. Conclusions

Lung compliance is significantly reduced during laparoscopic gynecological procedures due to pneumoperitoneum, as well as the Trendelenburg position. As shown in this study, despite the removal of these factors, the compliance remained 23.8% lower than the initial value. After the RM, compliance returned to the initial values. Specifically, it increased by 17.5% compared to the compliance during the induction of anesthesia.

According to data analysis, the RM was more beneficial in patients who had higher BMI, lower compliance, and higher Pplat at the induction of anesthesia. Specifically, according to the stratification analysis, the group of patients with a median BMI of 30.32 kg/m^2^ (IQR: 1.05 kg/m^2^) showed an 81.9% increase in compliance between time points T7 and T8. The group of patients with a higher Pplat at time T1 (median: 16.5 cmH_2_O; IQR: 0.75 cmH_2_O) showed similar results, as the corresponding increase in compliance after the RM was 79.4% (T7 vs. T8; *p* < 0.05). Finally, RM also had favorable results in patients with low initial median compliance (41.5 mL/cmH_2_O; IQR: 9.75 mL/cmH_2_O) as a 61.6% increase in compliance was observed between T7 and T8. Accordingly, the difference in the PaO_2_/FiO_2_ ratio between the two groups was not statistically significant at time T1. However, at time point T7, patients with lower baseline compliance showed statistically significantly lower median PaO_2_/FiO_2_ compared to those with higher baseline compliance (261.0, IQR: 52.8 and 363.0, IQR: 86.8, respectively). With the RM, this difference is reduced and ceases to be statistically significant. 

In conclusion, RM after desufflation is beneficial for restoring compliance and improving gas exchange. Further studies are necessary in order to compare different types of RMs and their efficacy, to investigate the effect of RMs in obese patients undergoing laparoscopic surgery, and to investigate the alternations of RMs and pulmonary mechanics concerning patients with pre-existing cardiopulmonary disease.

## Figures and Tables

**Table 1:** Demographic characteristics.

Demographic Characteristics	Median Values	IQR
Age (years)	44.0	12.0
ASA I	15 (48.4%)	
ASA II	16 (51.6%)	
Height (in cm)	164	6.5
Weight (in kg)	62.5	17.0
IBW (in kg)	56.0	6.01
BMI (kg/m^2^)	22.9	8.2

Values are presented as the median, IQR, or number (%). ASA, American Society of Anesthesiology. IBW: ideal body weight (kg). BMI: Body Mass Index (kg/m^2^).

**Table 2:** Average values of the procedure’s characteristics.

Procedure’s Characteristics	Median Values	IQR
Surgery duration (in minutes)	145.0	65.0
Anesthesia duration (in minutes)	175.0	67.5
Crystalloids (mL/kg/h)	10.6	3.0
Propofol (mg)	200	25.0
Rocuronium (mg)	40.1	6.0
Desflurane (% min)	1044.2	40.0

Values are presented as the median, IQR, or number (%).

## Data Availability

Data are contained within the article and Supplementary Materials.

## Supplementary Materials

The following supporting information can be downloaded at: https://www.mdpi.com/article/10.3390/jpm14050451/s1. Table S1: *p*-values (using Mann–Whitney U test) for the stratification analysis data for the effect of BMI on DP (A). PIP (B). Pplat (C) and compliance (D). Table S2: *p*-values (using Mann–Whitney U test) for the stratification analysis data for the effect of initial compliance (CdynT1) on Pplat (A). PIP (B). DP (C) and compliance (D). Table S3: *p*-values (using Mann–Whitney U test) for the stratification analysis data for the effect of initial DP (DP_T1) on DP (A). PIP (B). Pplat (C) and compliance (D). Table S4: *p*-values (using Mann–Whitney U test) for the stratification analysis data for the effect of initial Pplat (Pplat_T1) on Pplat (A). PIP (B). DP (C) and compliance (D). Table S5: *p*-values (using Mann–Whitney U test) for the initial dataset of 31 patients.

## References

[1] Levy L., Tsaltas J. (2021). Recent advances in benign gynecological laparoscopic surgery. Fac. Rev..

[2] Hottenrott S., Schlesinger T., Helmer P., Meybohm P., Alkatout I., Kranke P. (2020). Do Small Incisions Need Only Minimal Anesthesia? Anesthetic Management in Laparoscopic and Robotic Surgery. J. ClinMed..

[3] Oti C., Mahendran M., Sabir N. (2016). Anaesthesia for laparoscopic surgery. Br. J. Hosp. Med..

[4] Raźnikiewicz A., Korlacki W., Grabowski A. (2020). The role of laparoscopy in paediatric and adolescent gynaecology. Wideochir. Inne Tech. Maloinwazyjne.

[5] Wang D., Dong T., Shao Y., Gu T., Xu Y., Jiang Y. (2019). Laparoscopy versus open appendectomy for elderly patients, a meta-analysis and systematic review. BMC Surg..

[6] Sharma A., Dahiya D., Kaman L., Saini V., Behera A. (2016). Effect of various pneumoperitoneum pressures on femoral vein hemodynamics during laparoscopic cholecystectomy. Updates Surg..

[7] Alselaim N.A., Altoub H.A., Alhassan M.K., Alhussain R.M., Alsubaie A.A., Almomen F.A., Almutairi A.M., Bin Gheshayan S.F. (2022). The role of laparoscopy in emergency colorectal surgery. Saudi Med J..

[8] Cinnella G., Grasso S., Spadaro S., Rauseo M., Mirabella L., Salatto P., De Capraris A., Nappi L., Greco P., Dambrosio M. (2013). Effects of recruitment maneuver and positive end-expiratory pressure on respiratory mechanics and transpulmonary pressure during laparoscopic surgery. Anesthesiology.

[9] Rauseo M., Spadaro S., Mirabella L., Cotoia A., Laforgia D., Gaudino G., Vinella F., Ferrara G., Gattullo A., Tullo L. (2023). Electrical Impedance Tomography during Abdominal Laparoscopic Surgery: A Physiological Pilot Study. J. Clin. Med..

[10] SXue S., Wang D., Tu H.Q., Gu X.P., Ma Z.L., Liu Y., Zhang W. (2024). The effects of robot-assisted laparoscopic surgery with Trendelenburg position on short-term postoperative respiratory diaphragmatic function. BMC Anesthesiol..

[11] Miskovic A., Lumb A.B. (2017). Postoperative pulmonary complications. Br. J. Anaesth..

[12] Wang Y.H., Su P.C., Huang H.C., Au K., Lin F.C.F., Chen C.Y., Chou M.C., Hsia J.Y. (2023). Pulmonary Recruitment Prior to Intraoperative Multiple Pulmonary Ground-Glass Nodule Localization Increases the Localization Accuracy-A Retrospective Study. J. Clin. Med..

[13] Turan Civraz A.Z., Saracoglu A., Saracoglu K.T. (2023). Evaluation of the Effect of Pressure-Controlled Ventilation-Volume Guaranteed Mode vs. Volume-Controlled Ventilation Mode on Atelectasis in Patients Undergoing Laparoscopic Surgery: A Randomized Controlled Clinical Trial. Medicina.

[14] Holt P.R., Altayar O., Alpers D.H. (2023). Height with Age Affects Body Mass Index (BMI) Assessment of Chronic Disease Risk. Nutrients.

[15] El-Tahan M.R., Al Dossary N.D., El Emam H., Diab D.G., Al’Saflan A., Zien H., Al Ahmadey M., Deria A. (2012). Does hypocapnia before and during carbon dioxide insufflation attenuate the hemodynamic changes during laparoscopic cholecystectomy?. Surg. Endosc..

[16] Young C.C., Harris E.M., Vacchiano C., Bodnar S., Bukowy B., Elliott R.R.D., Migliarese J., Ragains C., Trethewey B., Woodward A. (2019). Lung-protective ventilation for the surgical patient: International expert panel-based consensus recommendations. Br. J. Anaesth..

[17] Liaqat A., Mason M., Foster B.J., Kulkarni S., Barlas A., Farooq A.M., Patak P., Liaqat H., Basso R.G., Zaman M.S. (2022). Evidence-Based Mechanical Ventilatory Strategies in ARDS. J. Clin. Med..

[18] Tsukamoto M., Goto M., Hitosugi T., Matsuo K., Yokoyama T. (2023). Comparison of the tidal volume by the recruitment maneuver combined with positive end-expiratory pressure for mechanically ventilated children. Sci. Rep..

[19] Santos R.S., Silva P.L., Pelosi P., Rocco P.R. (2015). Recruitment maneuvers in acute respiratory distress syndrome: The safe way is the best way. World J. Crit. Care Med..

[20] Gattinoni L., Collino F., Camporota L. (2024). Assessing lung recruitability: Does it help with PEEP settings?. Intensive Care Med..

[21] da Silva K., Oliveira C.C., Cabral L.F., Malaguti C., José A. (2023). Pulmonary expansion manoeuvres compared to usual care on ventilatory mechanics, oxygenation, length of mechanical ventilation and hospital stay, extubation, atelectasis, and mortality of patients in mechanical ventilation: A randomized clinical trial. PLoS ONE.

[22] Umbrello M., Chiumello D. (2018). Interpretation of the transpulmonary pressure in the critically ill patient. Ann. Transl. Med..

[23] Pei S., Wei W., Yang K., Yang Y., Pan Y., Wei J., Yao S., Xia H. (2022). Recruitment Maneuver to Reduce Postoperative Pulmonary Complications after Laparoscopic Abdominal Surgery: A Systematic Review and Meta-Analysis. J. Clin. Med..

[24] Cakmakkaya O.S., Kaya G., Altintas F., Hayirlioglu M., Ekici B. (2009). Restoration of pulmonary compliance after laparoscopic surgery using a simple alveolar recruitment maneuver. J. Clin. Anesth..

[25] Sümer I., Topuz U., Alver S., Umutoglu T., Bakan M., Zengin S.Ü., Coşkun H., Salihoglu Z. (2020). Effect of the “Recruitment” Maneuver on Respiratory Mechanics in Laparoscopic Sleeve Gastrectomy Surgery. Obes. Surg..

[26] Oh E.J., Lee E.J., Heo B.-Y., Huh J., Min J.-J. (2022). Physiological benefits of lung recruitment in the semi-lateral position after laparoscopic surgery: A randomized controlled study. Sci. Rep..

[27] Kung S.-C., Hung Y.-L., Chen W.-L., Wang C.-M., Chang H.-C., Liu W.-L. (2019). Effects of Stepwise Lung Recruitment Maneuvers in Patients with Early Acute Respiratory Distress Syndrome: A Prospective, Randomized, Controlled Trial. J. Clin. Med..

[28] Silva P.L., Moraes L., Santos R.S., Samary C., Ramos M.B.A., Santos C.L., Morales M.M., Capelozzi V.L., Garcia C.S.N.B., de Abreu M.G. (2013). Recruitment maneuvers modulate epithelial and endothelial cell response according to acute lung injury etiology. Crit. Care Med..

[29] Kang H., Yang H., Tong Z. (2019). Recruitment manoeuvres for adults with acute respiratory distress syndrome receiving mechanical ventilation: A systematic review and meta-analysis. J. Crit. Care..

[30] Patel S.K., Bansal S., Puri A., Taneja R., Sood N. (2022). Correlation of Perioperative Atelectasis With Duration of Anesthesia, Pneumoperitoneum, and Length of Surgery in Patients Undergoing Laparoscopic Cholecystectomy. Cureus.

[31] Monastesse A., Girard F., Massicotte N., Chartrand-Lefebvre C., Girard M. (2017). Lung Ultrasonography for the Assessment of Perioperative Atelectasis: A Pilot Feasibility Study. Anesth. Analg..

[32] Shanmugasundaram K., Bade G., Sampath M., Talwar A. (2023). Effect of Obesity on Airway Mechanics. Indian J. Endocrinol. Metab..

[33] Gong Y., Liu L., Zhang W. (2023). Pressure-controlled inverse ratio ventilation improves gas exchange in obese children undergoing laparoscopic surgery: A randomized controlled study. Am. J. Transl. Res..

[34] Braun M., Ruscher L., Fuchs A., Kämpfer M., Huber M., Luedi M.M., Riva T., Vogt A., Riedel T. (2023). Atelectasis in obese patients undergoing laparoscopic bariatric surgery are not increased upon discharge from Post Anesthesia Care Unit. Front. Med..

[35] Hansen B. (2021). Fluid Overload. Front. Vet. Sci..

[36] Virág M., Rottler M., Gede N., Ocskay K., Leiner T., Tuba M., Ábrahám S., Farkas N., Hegyi P., Molnár Z. (2022). Goal-Directed Fluid Therapy Enhances Gastrointestinal Recovery after Laparoscopic Surgery: A Systematic Review and Meta-Analysis. J. Pers. Med..

