# Supplementary material for: Restoration of Pulmonary Compliance after Laparoscopic Gynecologic Surgery Using a Recruitment Maneuver
Source: J Pers Med. 2024 Apr 25;14(5):451. doi: 10.3390/jpm14050451 (PMC11122417; doi:10.3390/jpm14050451)
Supplement: Supplementary file 1 [file jpm-14-00451-s001.zip › jpm-2938802-supplementary.pdf]

SUPPLEMENTARY MATERIAL

Table S1: P-values (using Mann-Whitney U Test) for the stratification analysis data for the effect of BMI on DP (A). PIP (B). Pplat (C) and Compliance (D)

**A. DP Data**									
		**T1**	**T2**	**T3**	**T4**	**T5**	**T6**	**T7**	**T8**
**MIN**	**Average**	7.30	7.40	11.80	12.60	14.00	11.30	8.50	6.80
	**STD**	1.95	2.07	1.40	2.22	2.49	4.19	1.96	1.55
**MAX**	**Average**	11.20	10.70	16.90	17.70	18.00	15.60	13.70	8.70
	**STD**	3.19	2.87	3.90	4.14	3.97	3.31	3.09	3.77
	**p-value**	0.031	0.025	0.0022	0.0003	0.014	0.0036	0.0073	**0.241**

**B. PIP Data**									
		**T1**	**T2**	**T3**	**T4**	**T5**	**T6**	**T7**	**T8**
**MIN**	**Average**	13.90	14.00	19.40	18.80	20.50	18.00	15.50	14.40
	**STD**	1.60	1.89	2.76	2.15	1.72	4.78	2.42	4.88
**MAX**	**Average**	18.20	17.10	23.70	24.20	25.00	22.70	21.50	15.20
	**STD**	3.29	2.85	4.22	5.59	4.88	3.50	3.24	3.74
	**p-value**	0.0022	0.0004	0.037	0.0017	0.0017	0.045	0.0003	**0.24`**

**C. Pplat Data**									
		**T1**	**T2**	**T3**	**T4**	**T5**	**T6**	**T7**	**T8**
**MIN**	**Average**	12.30	12.40	18.20	17.60	19.00	16.30	13.50	11.80
	**STD**	1.95	2.07	2.15	2.22	2.49	4.19	1.96	1.55
**MAX**	**Average**	16.20	15.30	21.60	21.90	23.00	20.60	18.70	13.70
	**STD**	3.19	2.83	3.78	4.79	3.97	3.31	3.09	3.77
	**p-value**	0.0006	0.0003	0.045	0.006	0.009	0.045	0.004	**0.062**

D. ComplianceData									
		**T1**	**T2**	**T3**	**T4**	**T5**	**T6**	**T7**	**T8**
**MIN**	**Average**	67.00	62.70	33.80	36.20	33.20	43.50	54.50	77.40
	**STD**	10.68	10.89	5.75	6.80	4.66	5.15	11.74	9.52
**MAX**	**Average**	47.20	46.80	27.00	26.20	27.00	31.50	37.80	65.60
	**STD**	11.81	12.80	4.94	7.93	6.20	6.13	8.02	15.20
	**p-value**	0.01	0.01	0.0046	0.009	0.025	0.03	0.021	**0.065**

Table S2: P-values (using Mann-Whitney U Test) for the stratification analysis data for the effect of initial Compliance (CdynT1) on Pplat (A). PIP (B). DP (C) and Compliance (D)

A. Pplat Data									
		**T1**	**T2**	**T3**	**T4**	**T5**	**T6**	**T7**	**T8**
**MIN**	**Average**	16.50	16.10	23.10	22.70	22.50	19.40	17.50	14.00
	**STD**	2.46	2.56	2.64	3.86	4.38	3.44	2.84	3.59
**MAX**	**Average**	11.60	12.00	17.60	17.70	19.40	17.20	14.00	12.00
	**STD**	1.17	0.82	2.12	1.64	3.24	4.59	3.77	2.05
	**p-value**	0.0018	0.0018	0.00029	0.0003	**0.054**	**0.795**	**0.089**	**0.168**

B. PIP Data									
		**T1**	**T2**	**T3**	**T4**	**T5**	**T6**	**T7**	**T8**
**MIN**	**Average**	17.70	17.60	25.40	25.00	24.50	21.10	20.20	15.10
	**STD**	2.63	2.50	2.88	4.62	4.74	3.63	2.94	3.63
**MAX**	**Average**	13.30	13.60	18.90	19.60	21.30	19.20	16.10	14.60

	**STD**	1.16	0.84	2.13	2.55	3.53	5.61	4.33	4.88
	**p-value**	0.0013	0.0013	0.0002	0.009	**0.09**	**0.2161**	**0.0531**	**0.734**

**C. DP Data**									
		**T1**	**T2**	**T3**	**T4**	**T5**	**T6**	**T7**	**T8**
**MIN**	**Average**	11.50	11.10	18.10	17.70	17.50	14.40	12.50	9.10
	**STD**	2.46	2.56	2.64	3.86	4.38	3.44	2.84	3.54
**MAX**	**Average**	6.60	7.00	13.30	12.70	14.40	12.20	9.00	7.00
	**STD**	1.17	0.82	1.49	1.64	3.24	4.59	3.77	2.05
	**p-value**	0.0001	0.0006	0.001	0.0016	**0.4219**	**0.125**	**0.0512**	**0.101**

**D. Compliance Data**									
		**T1**	**T2**	**T3**	**T4**	**T5**	**T6**	**T7**	**T8**
**MIN**	**Average**	42.10	43.70	25.00	25.00	25.60	34.10	36.80	57.50
	**STD**	5.99	12.37	4.47	5.33	6.52	8.50	7.51	8.17
**MAX**	**Average**	71.60	67.90	34.60	34.90	34.50	43.50	59.60	81.90
	**STD**	2.95	3.67	5.99	7.81	5.04	12.70	12.68	4.58
	**p-value**	0.0001	0.0004	0.0039	0.005	0.011	**0.25**	0.0035	0.0002

Table S3: P-values (using Mann-Whitney U Test) for the stratification analysis data for the effect of initial DP (DP_T1) on DP (A). PIP (B). Pplat (C) and Compliance (D).

**A. DP Data**									
		**T1**	**T2**	**T3**	**T4**	**T5**	**T6**	**T7**	**T8**

**MIN**	**Average**	6.10	6.70	12.70	11.50	13.30	11.20	8.00	6.80
	**STD**	0.74	0.67	1.25	2.37	2.67	4.42	1.94	2.10
**MAX**	**Average**	12.20	11.10	17.80	16.90	17.00	14.20	12.90	9.20
	**STD**	2.35	2.56	3.16	4.75	4.59	3.33	2.73	3.52
	**p-value**	0.00004	0.00075	0.00147	0.01373	0.06629	0.13806	0.00174	0.11286

**B. PIP Data**									
		**T1**	**T2**	**T3**	**T4**	**T5**	**T6**	**T7**	**T8**
**MIN**	**Average**	12.80	13.30	18.90	18.30	20.00	17.70	14.90	14.40
	**STD**	1.14	0.82	1.60	3.20	1.70	5.21	2.02	4.99
**MAX**	**Average**	18.50	17.50	25.00	24.00	24.10	20.80	20.10	15.10
	**STD**	2.59	2.64	3.59	5.60	4.95	3.49	2.92	3.63
	**p-value**	0.000192	0.001359	0.001188	0.026376	0.043421	0.192	0.0010	0.7913

**C. Pplat Data**									
		**T1**	**T2**	**T3**	**T4**	**T5**	**T6**	**T7**	**T8**
**MIN**	**Average**	11.10	11.70	17.70	16.50	18.30	16.20	13.00	11.80
	**STD**	0.74	0.67	1.25	2.37	2.67	4.42	1.94	2.10
**MAX**	**Average**	17.20	16.10	22.80	21.90	22.00	19.20	17.90	14.10
	**STD**	2.35	2.56	3.16	4.75	4.59	3.33	2.73	3.57
	**p-value**	0.00018	0.0002	0.0007	0.01073	**0.04629**	0.23806	0.0022	0.23020

**D. ComplianceData**									
		**T1**	**T2**	**T3**	**T4**	**T5**	**T6**	**T7**	**T8**
**MIN**	**Average**	68.90	65.30	34.80	37.90	34.10	45.70	59.10	78.90
	**STD**	6.87	7.57	5.81	8.16	4.75	12.25	12.63	6.77

**MAX**	**Average**	43.10	44.90	25.80	27.10	26.20	34.30	35.40	58.30
	**STD**	6.59	12.55	5.47	8.24	7.11	8.45	6.65	9.39
	**p-value**	0.00018	0.00290	0.008085	0.040898	0.009	0.027100	0.0018	0.0021

Table S4: P-values (using Mann-Whitney U Test) for the stratification analysis data for the effect of initial Pplat (Pplat_T1) on Pplat (A). PIP (B). DP (C) and Compliance (D).

A. Pplat Data

		**T1**	**T2**	**T3**	**T4**	**T5**	**T6**	**T7**	**T8**
**MIN**	**Average**	11.10	11.70	17.70	16.50	18.30	16.20	13.00	11.80
	**STD**	0.74	0.67	1.25	2.37	2.67	4.42	1.94	2.10
**MAX**	**Average**	17.20	16.40	22.60	21.70	22.40	19.80	17.90	14.40
	**STD**	2.35	2.59	3.34	4.88	4.17	3.01	2.73	3.31
	**p-value**	0.0002	0.0003	0.002593	0.031	0.014	**0.074090**	0.0033	**0.065**

B. PIP Data

		**T1**	**T2**	**T3**	**T4**	**T5**	**T6**	**T7**	**T8**
**MIN**	**Average**	12.80	13.30	18.90	18.30	20.00	17.70	14.90	14.40
	**STD**	1.14	0.82	1.60	3.20	1.70	5.21	2.02	4.99
**MAX**	**Average**	18.50	17.80	24.80	23.70	24.70	21.50	20.30	16.00
	**STD**	2.59	2.62	3.77	5.79	4.16	3.10	2.67	3.33
	**p-value**	0.00018	0.0092	0.0018	0.0423	0.007032	**0.1212**	0.003	**0.245**

C. DP Data

		**T1**	**T2**	**T3**	**T4**	**T5**	**T6**	**T7**	**T8**
**MIN**	**Average**	6.10	6.70	12.70	11.50	13.30	11.20	8.00	6.80
	**STD**	0.74	0.67	1.25	2.37	2.67	4.42	1.94	2.10
**MAX**	**Average**	12.20	11.40	17.60	16.70	17.40	14.80	12.90	9.50
	**STD**	2.35	2.59	3.34	4.88	4.17	3.01	2.73	3.24

	**p-value**	0.000189	0.00058	0.0089	0.0028	0.031	**0.1030**	0.00035	**0.058**

**D. ComplianceData**									
		**T1**	**T2**	**T3**	**T4**	**T5**	**T6**	**T7**	**T8**
**MIN**	**Average**	**68.90**	**65.30**	**34.80**	**37.90**	**34.10**	**45.70**	**59.10**	**78.90**
	**STD**	**6.87**	**7.57**	**5.81**	**8.16**	**4.75**	**12.25**	**12.63**	**6.77**
**MAX**	**Average**	**45.70**	**46.30**	**26.20**	**28.60**	**25.50**	**33.20**	**36.00**	**59.90**
	**STD**	**9.65**	**12.96**	**5.39**	**8.95**	**6.10**	**8.04**	**7.53**	**10.10**
	**p-value**	0.000439	0.00727	0.0127	0.0414	0.0044	0.001	0.0092	0.0008

Table S5: P-values (using Mann-Whitney U Test) for the initial dataset of 31 patients.

**Demographics**															
**Age**	**Height**	**Weight**	**IBW**	**BMI**	**Total Crystalloids**	**Total EPHE**	**Propofol (mg)**	**Surgery duration**	**Anesthesiaduration**						
0.98	0.45	0.94	0.36	0.68	0.09	0.64	0.53	0.58	0.45						
**T1**															
**MAP**	**HR**	**SpO2**	**Raw**	**PIP**	**Pplat**	**PEEP**	**DP**	**VT**	**Cdyn**	**RR**	**PaO2/FiO2**	**EtCO2**	**PaCO2**	**HCO3**	**pH**
0.66	0.62	0.06	0.75	0.64	0.57	1.00	0.57	0.35	0.37	0.08	0.0	0.98	0.77	0.07	0.07
**T2**															
**MAP**	**HR**	**SpO2**	**Raw**	**PIP**	**Pplat**	**PEEP**	**DP**	**VT**	**Cdyn**	**RR**	**PaO2/FiO2**	**EtCO2**	**PaCO2**	**HCO3**	**pH**
0.27	0.29	0.36	0.18	0.15	0.41	1.00	0.41	0.35	0.63	0.09	0.98	0.88	0.92	0.06	0.12
**T3**															
**MAP**	**HR**	**SpO2**	**Raw**	**PIP**	**Pplat**	**Peep**	**DP**	**VT**	**Cdyn**	**RR**	**PaO2/FiO2**	**EtCO2**	**PaCO2**	**HCO3**	**pH**

0.59	0.25	0.41	0.89	0.92	0.92	1.00	0.63	0.35	0.53	0.43	0.11	0.81	0.55	0.59	0.25
**T4**															
**MAP**	**HR**	**SpO2**	**Raw**	**PIP**	**Pplat**	**PEEP**	**DP**	**VT**	**Cdyn**	**RR**	**PaO2/FiO2**	**EtCO2**	**PaCO2**	**HCO3**	**pH**
0.94	0.34	0.30	0.81	0.94	0.89	1.00	0.89	0.48	0.66	0.52	0.55	0.55	0.89	0.07	0.25
**T5**															
**MAP**	**HR**	**SpO2**	**Raw**	**PIP**	**Pplat**	**PEEP**	**DP**	**VT**	**Cdyn**	**RR**	**PaO2/FiO2**	**EtCO2**	**PaCO2**	**HCO3**	**pH**
0.69	0.33	0.06	1.00	0.90	0.58	1.00	0.58	0.09	0.75	0.31	0.25	0.06	0.38	0.51	0.59
**T6**															
**MAP**	**HR**	**SpO2**	**Raw**	**PIP**	**Pplat**	**PEEP**	**DP**	**VT**	**Cdyn**	**RR**	**PaO2/FiO2**	**EtCO2**	**PaCO2**	**HCO3**	**pH**
0.50	0.89	0.40	0.68	0.51	0.54	1.00	0.54	0.23	0.42	0.61	0.24	0.20	0.25	0.98	0.95
**T7**															
**MAP**	**HR**	**SpO2**	**Raw**	**PIP**	**Pplat**	**Peep**	**DP**	**VT**	**Cdyn**	**RR**	**PaO2/FiO2**	**EtCO2**	**PaCO2**	**HCO3**	**pH**
0.95	0.21	0.16	0.76	0.47	1.00	1.00	1.00	0.23	0.61	0.37	0.37	0.52	0.37	0.31	0.62
**T8**															
**MAP**	**HR**	**SpO2**	**Raw**	**PIP**	**Pplat**	**PEEP**	**DP)**	**VT**	**Cdyn**	**RR**	**PaO2/FiO2**	**EtCO2**	**PaCO2**	**HCO3**	**pH**
0.17	0.39	0.06	0.98	0.53	0.55	1.00	0.51	0.53	0.45	0.06	0.31	0.09	0.48	0.18	0.27
